# More autosomal dominant SPG18 cases than recessive? The first AD‐SPG18 pedigree in Chinese and literature review

**DOI:** 10.1002/brb3.2395

**Published:** 2021-11-03

**Authors:** Shuai Chen, Jin‐Long Zou, Shuang He, Wei Li, Jie‐Wen Zhang, Shu‐Jian Li

**Affiliations:** ^1^ Department of Neurology Zhengzhou University People's Hospital (Henan Provincial People's Hospital) Zhengzhou China; ^2^ Department of Neurology Henan University People's Hospital Zhengzhou China

**Keywords:** ERLIN2, gene, hereditary spastic paraplegia, SPG18

## Abstract

**Objective:**

Hereditary spastic paraplegia (HSP) due to *ERLIN2* gene mutations was designated as spastic paraplegia 18 (SPG18). To date, SPG18 families/cases are still rarely reported. All early reported cases shared the autosomal recessive (AR) inheritance pattern. Over the past 3 years, autosomal dominant (AD) or sporadic SPG18 cases had been continuously reported. Here, we reported the clinical and genetic features of the first autosomal dominant SPG18 pedigree in Chinese.

**Methods:**

We conducted detailed medical history inquiry, neurological examinations of the proband and his family members, and charted the family tree. The proband underwent brain and cervical magnetic resonance imaging (MRI), electromyography (EMG), and whole exome sequencing. Sanger sequencing was performed to verify the genetic variation in the proband and some family members. A literature review of all reported SPG18 families/cases was carried out to summarize the clinical‐genetic characteristics of SPG18 under different inheritance patterns.

**Results:**

Four patients were clinically diagnosed as chronic spastic paraplegia in three consecutive generations with the autosomal dominant inheritance model. All the patients presented juvenile‐adolescent onset and gradually worsening pure HSP phenotype. Clinical phenotypes were consistent within the family. Whole exome sequencing in the proband identified a previously reported heterozygous c.502G > A (p.V168M) mutation in exon 8 of *ERLIN2* gene. This mutation was cosegregated with the phenotype in the family and was classified as likely pathogenic according to American College of Medical Genetics and Genomics (ACMG) guidelines. To date, eight AR‐SPG18 families, five AD‐SPG18 families, and three sporadic cases had been reported. Clinical phenotype of AD‐SPG18 was juvenile‐adolescent onset pure HSP, while the phenotype of AR‐SPG18 was mostly complicated HSP with earlier onset and more severe conditions. In rare cases, the initial spastic paraplegia could evolve to rapidly progressive amyotrophic lateral sclerosis (ALS).

**Conclusions:**

We reported the first autosomal dominant SPG18 pedigree in Chinese Han population, which added more pathogenic evidence for V168M mutation. As more SPG18 cases reported, the essentials of SPG18 need to be updated in clinical practice. Special attentions should be given in gene test for upper motor neuron disorders in case of missing heterozygous mutations in *ERLIN2*.

## Graphical abstract

we reported the first autosomal dominant SPG18 pedigree in Chinese Han population, which added more pathogenic evidence for V168M mutation. The clinical phenotypes of SPG18 are expanded and are highly heterogeneous under different inheritance patterns. As more cases reported, the essentials of SPG18 need to be updated in clinical practice and genetic testing. Special attentions should be given in genetic testing of upper motor neuron disorders in case of missing heterozygous mutations in ERLIN2.

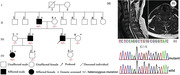

## 1 INTRODUCTION

Hereditary spastic paraplegias (HSPs) are a group of neurodegenerative upper motor neuron disorders that are clinically and genetically heterogeneous. The main clinical manifestations are gait abnormalities and spasticity in the lower extremities. Neurological examinations often revealed upper motor neuron signs including increased muscle tone, hyperreflexia, and pathologic reflexes. Clinically, HSP can be divided into the pure form and complicated form according to additional neurological and extraneurological signs. By modes of inheritance, HSP can be classified as autosomal dominant (AD), autosomal recessive (AR), X‐linked recessive (XLR), and mitochondrial maternal HSP. To date, more than 80 HSP gene loci have been reported. Generally, AD‐HSP is the most common type which mainly presented with pure HSP, while AR‐HSP mostly present with the complicated form of HSP. Interestingly, few HSPs can be inherited in both dominant and recessive patterns, including SPG3A, SPG7, SPG9, SPG18, SPG30, and SPG72 (Boutry et al., 2019). According to OMIM and a recent review, 19 of the 83 SPG loci presented with the AD inheritance pattern, 54 loci with the AR inheritance pattern, four loci with AD/AR inheritance pattern, and six loci with the XLR inheritance pattern (Saputra & Kumar, 2021).

The causative gene for SPG18 is endoplasmic reticulum lipid raft associated protein 2 gene (*ERLIN2*). *ERLIN2* was initially mapped in an autosomal recessive Turkish family with intellectual disability, motor impairment, and multiple joint contractures in 2011 (Yildirim et al., 2011). At the same time, a deletion mutation of *ERLIN2* was also identified in an autosomal recessive family with the complicated form of HSP in Saudi Arabia, which was designated as SPG18 (Alazami et al., 2011). Early reported families were all recessively inherited, complicated HSP (Alazami et al., 2011; Wakil et al., 2013; Yildirim et al., 2011). In 2018, two AD‐HSP families caused by heterozygous *ERLIN2* missense mutations were first reported in Norwegian populations (Rydning et al., 2018). So far, SPG18 families/cases reported were still limited. With more SPG18 cases discovered, clinical phenotypes also expanded. In addition to HSP, the HSP‐ALS conversion phenotype and primary lateral sclerosis phenotype were also seen (Al‐Saif et al., 2012; Amador et al., 2019).

Here, we reported the clinical and genetic features of the first autosomal dominant SPG18 pedigree in Chinese. We also summarized all the reported SPG18/*ERLIN2* families/cases to analyze the clinical and genetic features of SPG18 under different inheritance patterns.

## 2 SUBJECTS AND METHODS

### 2.1 Participants

**FIGURE 1:**
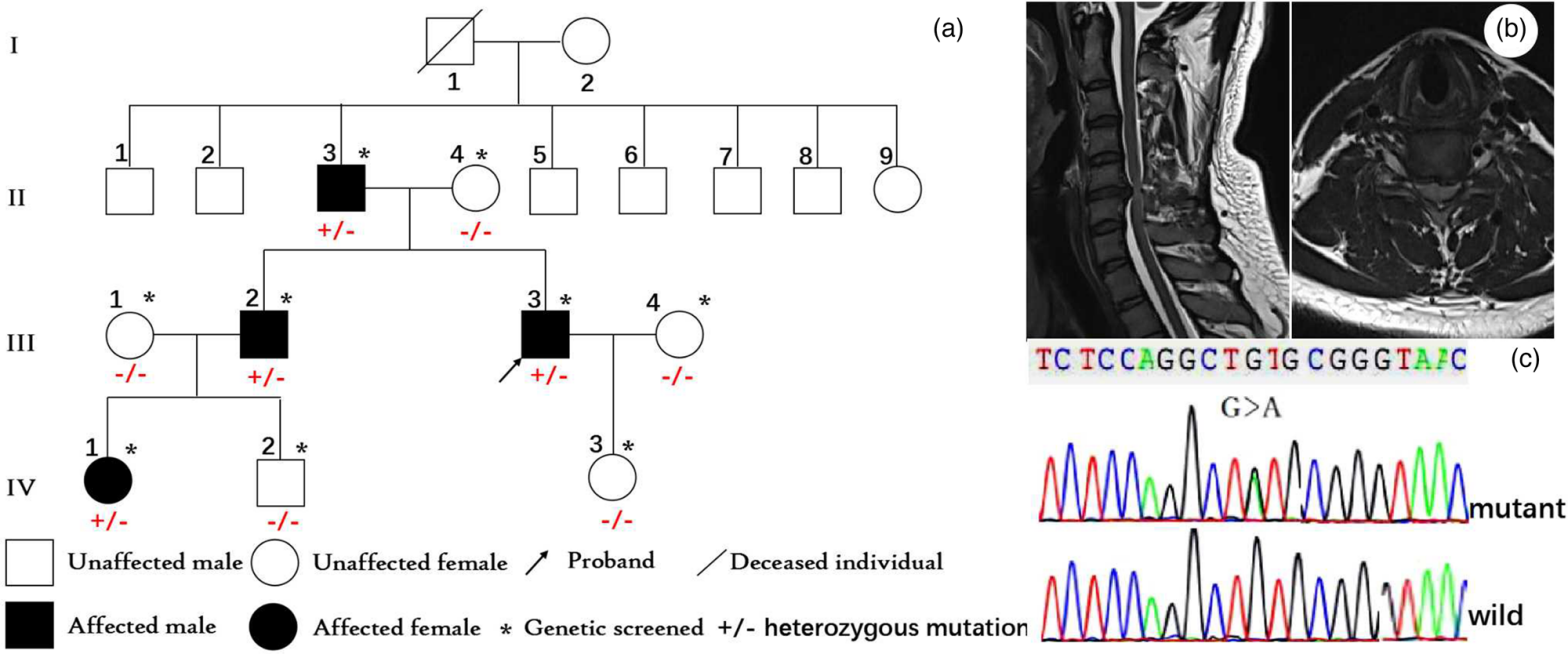
The Chinese Han autosomal dominant spastic paraplegia 18 (SPG18) pedigree. Family tree (a). Cervical spine magnetic resonance imaging (MRI) showed spinal canal stenosis and spinal cord degeneration at the C4/5 and C5/6 levels due to disc herniations (b). The V168M mutation by Sanger sequencing (c)

The proband was admitted to the People's Hospital of Zhengzhou University in 2018. We conducted detailed history inquiry and neurological examinations in the proband and some family members and drew the pedigree tree (Figure 1a). Genetic testing was performed in the proband and some family members. The study was approved by the ethics committee of People's Hospital of Zhengzhou University. Written informed consent was obtained from all study participants.

### 2.2 Methods

The proband underwent blood tests for blood routine, serum liver and kidney function, lipids, vitamin B12 and folate levels, vitamin E, serum copper, ceruloplasmin, ammonia, lactate, and cortisol levels. Brain and cervical‐thoracic spinal magnetic resonance imaging (MRI) scans were performed at a 3.0T magnetic resonance scanner (Siemens Skyra 3.0T). Nerve conduction velocity (NCV), needle electromyography, and somatosensory evoked potentials (SEP) were examined to detect lower motor neuron damages.

Peripheral blood was obtained from the proband (III3) and some family members (II3, II4, III1‐4, and IV1‐3) for genomic DNA extraction. Whole exome sequencing and multiplex ligation‐dependent probe amplification (MLPA) for *SPAST* and *ATL1* deletion/duplications and mitochondrial 16K screening were performed in the proband. Protein‐coding exome enrichment was performed by xGen Exome Research Panel v1.0 (IDT, Iowa, USA), which consists of 429,826 individually synthesized and quality‐controlled probes. High‐throughput sequencing was performed by Illumina NovaSeq 6000 series sequencer (PE150). The single‐nucleotide variants (SNV) and insertions and deletions (InDels) were performed by the GATK software (Genome Analysis ToolKit). Variants were filtered out with a minor allele frequency >0.5% according to the databases including gnomeAD, ExAC, and 1000 Genomes Project. The ANNOVAR software was used to annotate the functions of these variations. Functional prediction of candidate variations was analyzed by SIFT, Polyphen‐2, and Mutation Taster software. To improve the diagnostic efficiency, a phenotype‐driven designing virtual panel was generated by Mingjian software (Running Gene Inc, China). Mingjian is a self‐updating supportive diagnostic system based on the databases of HPO, OMIM, and HGMD. Core phenotype of the family was extracted and inputted into the software to generate a virtual panel of phenotype‐associated genes. The virtual panel was then used to filter and re‐annotate the candidate mutations (Wang et al., 2019). All variants were interpreted according to the ACMG recommendations. Genetic variations detected in whole exome sequencing were verified by Sanger sequencing.

## 3 RESULTS

### 3.1 Clinical presentation

The proband (III3) was a 30‐year‐old male farmer. He was admitted for the progressive stiffness and weakness of both lower limbs over the past 20 years. At childhood, he was noted with lower limbs weakness and abnormal gait. These symptoms gradually worsened. When he was 20, he could not run or walk fast due to leg stiffness. He was unable to flex his lower limbs freely, but was still able to walk independently. There were no abnormalities in upper limbs, no dysphagia, and no bowel and bladder disturbances. Past history and birth history were unremarkable. Neurological examinations revealed hyperreflexia in upper limbs (3+) and lower limbs (4+), muscle strength of grade 3 (Medical Research Council Scale) in lower limbs, pathological reflexes, and scissoring gait. Cranial nerves and peripheral nerves were intact. Cognition was normal with the Mini‐mental State Examination (MMSE) score of 28. Brain MRI showed no abnormalities. Cervical spinal MRI showed spinal canal stenosis and spinal cord degeneration at the C4/5 and C5/6 levels due to disc herniations (Figure 1b). MRI of the thoracic spine showed no spinal cord thinning. Nerve conduction and needle electromyography of the four limbs showed no abnormalities.

The proband's grandfather (I1) died at the age of 48 due to cerebral hemorrhage and had no walking abnormalities during his lifetime. The proband's grandmother (I2) was 90 years old without walking difficulties. The proband's father (II3) was 63 years old. Abnormal walking was noted when he was 15. He felt leg stiffness when he was 20. He could not run at the age of 35. Since the age of 50, he could not walk on his own and needed the assistance of crutches. Neurological examinations for the proband's father revealed lower limbs spasticity, hyperreflexia, and pathologic reflexes. Electromyography (EMG) for the proband's father showed no lower motor neuron impairment. The proband's father had two elder brothers, four younger brothers, and one younger sister, all of whom reported no walking abnormalities. The proband had one elder brother (III2) who presented frequent falls at the age of 8. His walk got worse during cold days. After the age of 25, he was not competent for fast walking and running. He could still walk slowly on his own, but had difficulties in flexing his feet. Lower limb spastic paraplegia signs were also noted. The proband's brother had a 10‐year‐old daughter (IV1) who presented with similar walking difficulties since the age of 8 and a 6‐year‐old son (IV2) who was normal. The proband had a 10‐year‐old daughter (IV3) with normal walking.

### 3.2 Genetic findings

All the patients within the family presented with juvenile‐adolescent onset, gradually aggravated pure HSP phenotype with the probable autosomal dominant inheritance. Whole exome sequencing (WES) was performed on the proband with an average sequencing depth of 120× and 99.45% coverage above 30×. A heterozygous c.502G > A mutation (p.V168M) in exon 8 of *ERLIN2* gene was identified (NM_007175.6; NP_009106.1) (Figure 1c). MLPA and mitochondrial 16K testing found no clinically significant genetic variations.

The V168M variant was not present in the Chinese Millionome Database (CMDB) containing over 5000 Chinese samples (http://cmdb.bgi.com/cmdb/). This mutation was also not present in the normal controls in gnomeAD, ExAC, and 1000 Genomes Project databases. In gnomeAD v2 database, there were 4900 East Asian controls. Sanger sequencing further confirmed the mutation in the proband and symptomatic family members of II3, III2, and IV1. Genetic mutation and phenotype in the family were cosegregated. This V168M mutation has been reported in an autosomal dominant French family with the HSP‐ALS phenoconversion (Amador et al., 2019). As seen in that article, functional predictions of the mutation by SIFT, Polyphen‐2, and Mutation Taster were all damaging and CADD score was 34. The valine amino acid is highly conserved across multiple species. Finally, this V168M mutation was classified as likely pathogenic according to ACMG guidelines (PS4, PM2, PP1, PP3, and PP4).

**FIGURE 2:**
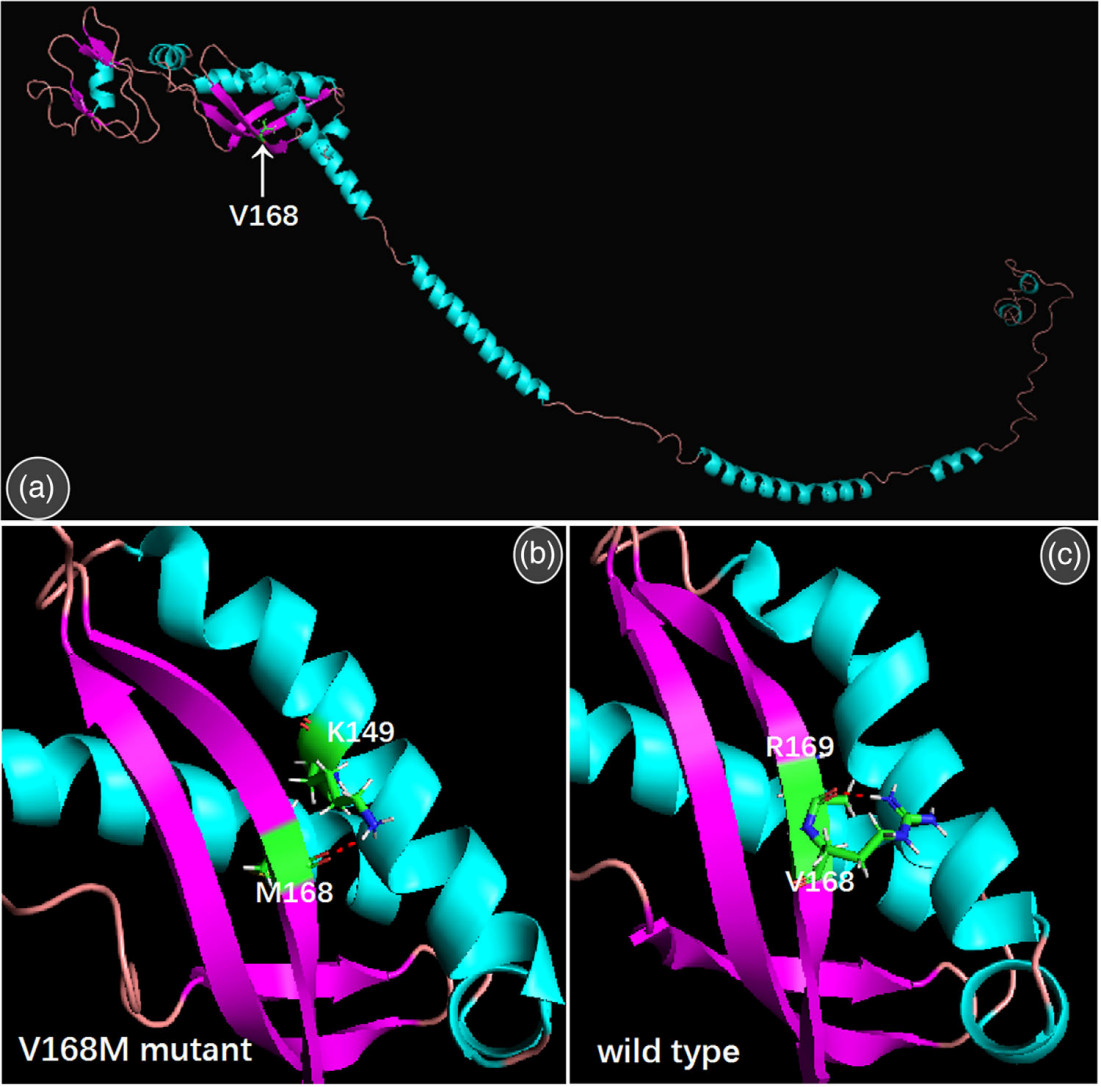
The predicted structures of ERLIN2. The structures of wild type ERLIN2 predicted by I‐TASSER on‐line Server. The white arrow indicates the position of 168th amino acid (a). The red dashed line indicates the hydrogen bond formed by V168 and neighboring R169 residues in wild type ERLIN2 (b). A new hydrogen bond formed by M168 and K149 residue from adjacent α‐helix, which slightly changed the β‐sheet structure where the 168th amino acid is located (c)

As there is no available protein structure of ERLIN2, full‐length amino acid sequences of erlin2 were used to predict the tertiary structure by online sever I‐TASSER (Yang et al., 2015). Multiple alignment templates in I‐TASSER contain the SPFH domain, but with identity less than 20%. Compared with the wild type, the tertiary structure of the mutant protein does not change largely. In wild‐type ERLIN2, the V168 and neighboring R169 residues form the hydrogen bond. But in the mutant protein, M168 and K149 residue from adjacent α‐helix form an alternative hydrogen bond. (Figure 2).

### 3.3 Summary of SPG18 families/cases

To date, eight AR‐SPG18 families, five AD‐SPG18 families, and three sporadic cases have been reported (Alazami et al., 2011; Al‐Saif et al., 2012; Amador et al., 2019; Park et al., 2020; Rydning et al., 2018; Srivastava et al., 2020; Tian et al., 2016; Wakil et al., 2013; Yildirim et al., 2011) (Table 1). Patients were distributed in Middle East, Turkey, China, Korea, France, and Norway. Most of the AR‐SPG18 families had consanguineous marriages (6/8). Seven AR‐SPG18 families harbored the homozygous gene variants including large deletions, insertions, duplications, splicing mutations, and point mutations. One AR‐SPG18 family from China harbored the compound heterozygous mutations. Of the eight AR‐SPG18 families, six families had the disease onset before 2 years of age, and the other two families were adolescent onset. Clinical phenotypes of AR‐SPG18 were dominated by complicated HSP (7/8). Only one Chinese family with the compound heterozygous mutations presented as pure HSP. Other superimposed manifestations include limb deformities, language and intellectual impairment, epilepsy, lower motor neuron signs, and deafness.

**TABLE 1:** A summary of all reported spastic paraplegia 18 (SPG18) families/cases

	Consanguineous marriage	Inheritance pattern	Onset age (M: month; Y: year)	Phenotype	Other features	Genetic variants	Reference
F1	Yes	AR	30 M	Complicated	Language and intellectual impairment; epilepsy; limb deformities	Homozygous 20 kb deletions causing loss of exon1and mislocalization of exon 2 in *ERLIN2* (null mutation)	Alazami et al. (2011)
F2	Yes	AR	6 M‐2 Y	Complicated	Language problems; joint contracture; seizure	Homozygous c.812_813insac (N272Pfs*4)	Yildirim et al. (2011)
F3	Yes	AR	8 M	Complicated (primary lateral sclerosis)	Limb deformities; language problems; joint contracture	Homozygous c.499‐1G > T (p.Q169Lfs*4)	Al‐Saif et al. (2012)
F4	Yes	AR	1 Y	Complicated	Cognitive and speech problems	Homozygous c.499‐1G > T (p.Q169Lfs*4)	Wakil et al. (2013)
F5	–	AR	39 Y	Pure		Compound heterozygous c.538C > T (R180C) and c.298 + 1G > T	Tian et al. (2016)
F6	–	AR	15–20 Y	Complicated	Conversion to ALS (38 years later); intellectual impairment	Homozygous c.899A > T (D300V)	Amador et al. (2019)
F7	Yes	AR	8 M	Complicated	Deafness; intellectual impairment; language problems; movement disorders	Homozygous c.861_874dup14 (n292rfsx26)	Srivastava et al. (2020)
F8	Yes	AR	1–2 Y	Complicated	Cognitive and language problems; movement disorders	Homozygousc.861_874dup14 (n292rfsx26)	Srivastava et al. (2020)
F9	–	AD	13–46 Y	Pure		Heterozygous c.386G > C (S129T)	Rydning et al. (2018)
F10	–	AD	9–28 Y	Pure		Heterozygous c.386G > C (S129T)	Rydning et al. (2018)
F11	–	AD	25–45 Y	Pure	Conversion to ALS (25–30 years later)	Heterozygous c.502G > A (V168M)	Amador et al. (2019)
F12	–	AD	15–48 Y	Pure		Heterozygous c.452 C > T (A151V)	Park et al. (2020)
F13	–	AD	8—15 Y	Pure		Heterozygous c.502G > A (V168M)	Current case
S1	–	sporadic	20 Y	Pure	Conversion to ALS (45 years later)	Heterozygous c.374A > G (N125S(	Amador et al. (2019)
S2	–	sporadic	32 Y	Pure		Heterozygous c.187C > A (Q63K)	Srivastava et al. (2020)
S3	–	sporadic	2 Y	Pure	Language problems; movement disorders	Homozygous c.407T > G (V136G)	Srivastava et al. (2020)

Abbreviation: ALS, amyotrophic lateral sclerosis.

Among the five AD‐SPG18 families, age at onset ranged from 8 to 48 years, which was older than that in AR‐SPG18 families. The clinical phenotypes in the five families were mainly pure HSP with long survival, and the mutations were all heterozygous mutations. In rare cases, the initial spastic paraplegia could evolve to rapidly progressive amyotrophic lateral sclerosis (ALS) (Amador et al., 2019).

Clinical phenotypes of the three sporadic cases were also pure HSP. Two cases had the heterozygous mutations. Another case had the homozygous missense mutation due to uniparental disomy and presented an earlier onset at 2 years of age (Srivastava et al., 2020).

## 4 DISCUSSION

Since 2011, HSP families/cases caused by *ERLIN2* gene mutations have been occasionally reported. The initial cases were basically complicated AR‐HSPs. In 2018, pure AD‐HSP families were first reported in the Norwegian population (Rydning et al., 2018). In the present study, we reported the first AD‐SPG18 family with the V168M mutation in Chinese. The cervical spinal MR in the proband showed the spinal cord compression due to herniated cervical disc. He was initially diagnosed as cervical spondylotic myelopathy. After inquiring the family history, an alternative diagnosis of HSP was proposed. It was confusing to determine whether his spastic paraplegia symptoms were caused by cervical spondylosis or by *ERLIN2* gene mutation. The symptoms of the proband may be best explained by dualism. But the following points supported gene mutations may be the major reason. Symptoms of the proband began at childhood, while cervical spondylosis was generally rare at childhood. Besides, the proband, his brother, and his father all presented chronic spastic paraplegia and carried the same genetic mutation. But cervical spine MR of his brother and father were normal. The needle EMG and somatosensory evoked potentials (SEP) examinations in the proband revealed no abnormalities either. Also, it is hard to clarify the relationship between cervical spondylosis and SPG18.

Compared with the first V168M family in France, the current family had a younger onset age (8–15 years), while the French family had the onset age between 25 and 45 years of age. The initial phenotype of the French family was pure HSP, but after 20–30 years, the phenotype converted to rapidly progressive ALS. All patients in the current family had the pure HSP phenotype. The oldest patient (II3) was 63 years old, whose EMG showed no ALS related abnormalities. The follow‐up of the HSP‐ALS phenoconversion was warranted.

There are few HSPs with both the dominant and recessive inheritance models, including SPG3A, SPG7, SPG9, SPG18, SPG30, and SPG72 (Boutry et al., 2019). In the HSPs with mixed inheritance patterns, distributions of AD/AR patterns are also different. For example, SPG3A is mainly dominantly inherited, while SPG7 is mainly recessively inherited. In some conditions, the cases reported are too few to summarize the distributions of AD/AR patterns. Based on the limited cases, the inheritance patterns of SPG18 are complex (recessive, dominant, and sporadic). Recent reported cases were all AD‐SPG18 and sporadic SPG18, which reminds us not to filter out heterozygous mutations in *ERLIN2* in genetic testing.

Clinical phenotypes of AR‐SPG18 were mostly complicated HSP with earlier onset and severe conditions. AR‐SPG18 gene variants mostly led to ERLIN2 expression defects or loss of function, which may explain the severe clinical picture in AR‐SPG18 (Al‐Saif et al., 2012). The physiological function of ERLIN2 protein is not fully understood yet. It forms an erlin1/2 complex with the homologous erlin1 protein. One well‐defined function of this complex is to recruit RNF170, a ubiquitin ligase (E3), to the activated inositol 1,4,5‐trisphosphate receptor (IP3R), then ubiquitylate and degrade them (Browman et al., 2006; Lu et al., 2011; Wright et al., 2015). Interestingly, mutations in the *ERLIN1* and *RNF170* gene could also cause HSP (Wagner et al., 2019). Three AR‐SPG62/*ERLIN1* families were reported presenting with the pure HSP with an onset age of 1–13 years old. Another SPG62/*ERLIN1* family had the phenotype of juvenile onset, recessively inherited HSP. But the phenotype converted to slowly progressive ALS at 50–60 years of age (Tunca et al., 2018). Similarly, rare SPG18/*ERLIN2* cases also presented the HSP‐ALS phenoconversion (Novarino et al., 2014). *RNF170* gene mutations lead to AR‐HSPs. The clinical features of the four reported *RNF170*‐HSP families were infantile onset, complicated HSP with varying degrees of optic atrophy and cerebellar ataxia (Novarino et al., 2014). Consistently, inheritance patterns and clinical presentations of *ERLIN1*, *ERLIN2*, and *RNF170*‐related diseases were all heterogeneous.

In conclusion, we reported the first autosomal dominant SPG18 pedigree in Chinese Han population, which added more pathogenic evidence for V168M mutation. The clinical phenotypes of SPG18 are expanded and are highly heterogeneous under different inheritance patterns. As more cases reported, the essentials of SPG18 need to be updated in clinical practice and genetic testing. Special attention should be given in genetic testing of upper motor neuron disorders in case of missing heterozygous mutations in *ERLIN2*.

## CONFLICT OF INTEREST

The authors declare no conflict of interest.

## AUTHOR CONTRIBUTIONS

Shuai Chen and Shu‐Jian Li conceived the idea. Jie‐Wen Zhang and Shuang He collected the clinical and genetic data. Shuai Chen contributed to the data interpretation and writing of the manuscript. The revision of the manuscript was done by Shu‐Jian Li and Jie‐Wen Zhang. All authors approved the submission of the paper.

### TRANSPARENT PEER REVIEW

The peer review history for this article is available at https://publons.com/publon/10.1002/brb3.2395

## Data Availability

The data are available via contacting corresponding author, Shu‐jian Li.
